# Pre-stroke Physical Activity and Cerebral Collateral Circulation in Ischemic Stroke: A Potential Therapeutic Relationship?

**DOI:** 10.3389/fneur.2022.804187

**Published:** 2022-02-15

**Authors:** Stanley Hughwa Hung, Sharon Kramer, Emilio Werden, Bruce C. V. Campbell, Amy Brodtmann

**Affiliations:** ^1^The Florey Institute of Neuroscience and Mental Health, The University of Melbourne, Melbourne, VIC, Australia; ^2^Centre for Quality and Patient Safety Research, Alfred Health Partnership, Melbourne, VIC, Australia; ^3^Faculty of Health, School of Nursing and Midwifery, Deakin University, Geelong, VIC, Australia; ^4^Melbourne Dementia Research Centre, The Florey Institute of Neuroscience and Mental Health, Parkville, VIC, Australia; ^5^Department of Medicine and Neurology, Melbourne Brain Centre at the Royal Melbourne Hospital, University of Melbourne, Parkville, VIC, Australia

**Keywords:** physical activity, exercise, collateral circulation, stroke, recovery, hypothesis

## Abstract

Favorable cerebral collateral circulation contributes to hindering penumbral tissue from progressing to infarction and is associated with positive clinical outcomes after stroke. Given its clinical importance, improving cerebral collateral circulation is considered a therapeutic target to reduce burden after stroke. We provide a hypothesis-generating discussion on the potential association between pre-stroke physical activity and cerebral collateral circulation in ischemic stroke. The recruitment of cerebral collaterals in acute ischemic stroke may depend on anatomical variations, capacity of collateral vessels to vasodilate, and individual risk factors. Physical activity is associated with improved cerebral endothelial and vascular function related to vasodilation and angiogenic adaptations, and risk reduction in individual risk factors. More research is needed to understand association between cerebral collateral circulation and physical activity. A presentation of different methodological considerations for measuring cerebral collateral circulation and pre-stroke physical activity in the context of acute ischemic stroke is included. Opportunities for future research into cerebral collateral circulation, physical activity, and stroke recovery is presented.

## Introduction

In ischemic stroke, rapid access to targeted treatment is essential to prevent disability and mortality (1, 2). Prolonged blood flow disruption as a result of vessel occlusions or stenosis can result in permanent brain tissue damage (i.e., ischemic core) (3). Hypoperfused, electrically inactive brain tissue surrounding the ischemic core, known as the ischemic penumbra, can potentially be salvaged or progress to infarction (2, 4). However, the progression of ischemic core growth is highly variable (3). One important factor is the level of perfusion in the penumbra (2). Even when reperfusion is not achieved, the ischemic core does not always grow to the full extent of the vascular territory affected (5). The cerebral collateral circulation system is recognized as an underlying factor that determines the level of perfusion in the penumbra and associated ischemic core growth in ischemic stroke (6). The level of perfusion offered by the collateral circulation has clinical implications. Leng et al. conducted a systematic review and meta-analysis examining 35 studies on the association between cerebral collateral circulation and the efficacy and safety of endovascular treatments (7). The authors reported that favorable collateral circulation was associated with higher rates of favorable functional outcomes at 3-months post-stroke, reduced risk of symptomatic intracranial hemorrhage during endovascular treatments, and reduced risk of mortality at 3-months (7). Other investigators have further reported that unfavorable collateral circulation is associated with many clinically important stroke outcomes, including faster infarct growth, larger final infarct volumes, proximal occlusions, higher stroke severity on admission, and poorer functional outcomes after stroke (8–12).

Given the established link between favorable collateral circulation and improved stroke outcomes, the cerebral collateral circulation system is regarded as a potential therapeutic target to improve outcomes in acute ischemic stroke (6, 13). Pharmaceutical and non-pharmaceutical strategies to optimize collateral circulation in the acute stroke setting have been proposed, including optimal head position during acute care (14, 15), medications to induce blood volume expansion, vasodilation or hypertension, neural stimulation, partial occlusion of the aorta, and limb compression devices (6, 13). However, the most appropriate strategies remain unclear (6). Increasing physical activity, among other lifestyle factors, may also be a potential strategy to optimize pre-stroke collateral circulation (16). Pre-stroke physical activity has been associated with lower admission stroke severity, reduced infarct volume, lower risk of mortality, and favorable functional outcomes after stroke (17–23). Yet our understanding of the association between pre-stroke physical activity and collateral circulation in acute ischemic stroke is limited.

The purpose of the current article is to provide a hypothetical basis for and discussion of the association between pre-stroke physical activity and collateral circulation. Specifically, we will (1) describe the collateral circulation system and factors influencing collateral circulation recruitment, (2) describe physical activity and the potential association between pre-stroke physical activity and collateral circulation, (3) discuss how collateral circulation and pre-stroke physical activity can be measured and (4) discuss future directions for the potential utility of collateral circulation and physical activity assessment in secondary prevention and recovery after stroke.

## Search Strategy

For this hypothesis and theory review, we searched the PubMed electronic database and Google Scholars for peer-reviewed journal article. The search was conducted using appropriate Boolean connectors where applicable, including individual or combined keywords and concepts associated with physical activity, exercise, collateral circulation, and ischemic stroke. Reference lists of relevant studies were hand searched. Relevant human and animal studies were included. No date restrictions were applied.

## The Cerebral Collateral Circulation System

**Figure 1:**
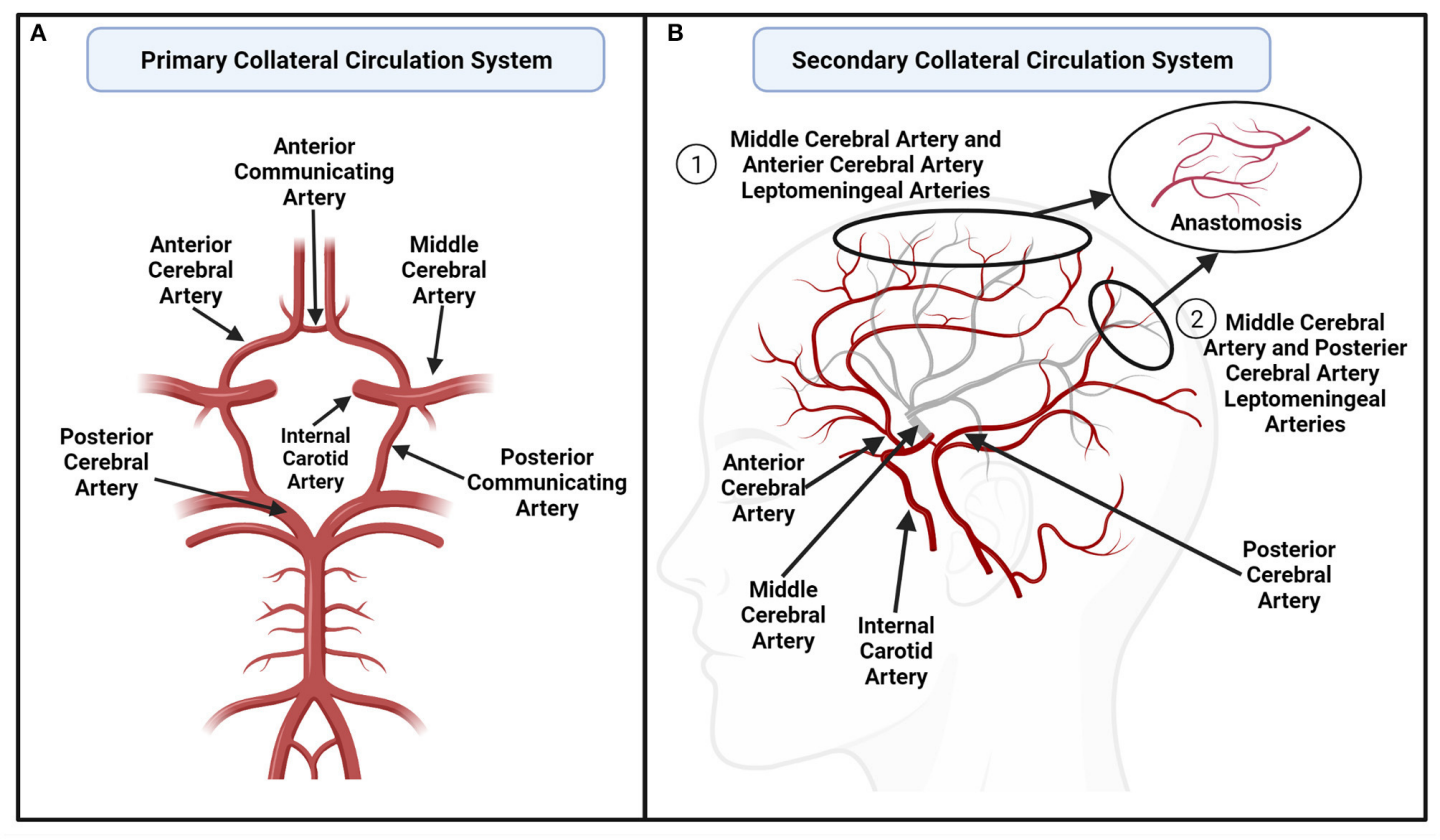
**(A)** An illustration of the primary collateral circulation system, including the individual arteries of the Circle of Willis. The left and right anterior cerebral arteries (ACA) are connected by the anterior communicating artery, while the middle cerebral arteries (MCA) and posterior cerebral arteries (PCA) are connected by the posterior communicating arteries (25). **(B)** A sagittal, transparent view of selected right cerebral arteries to illustrate an example of the secondary collateral circulation system, where the MCA (gray arteries) forms leptomeningeal arterial anastomoses with the (1) ACA and (2) PCA. Created with BioRender.com.

The cerebral collateral circulation system refers to complex networks of supplementary blood vessels that are recruited to provide alternative routes to maintain adequate cerebral blood flow when primary blood vessels are obstructed (24). These supplementary blood vessels are particularly important in ischemic stroke, where inadequate arterial blood flow results from vessel narrowing (i.e., stenosis) or embolism (i.e., occlusion). The anatomical structures of the cerebral collateral circulation system are complex, and can be subdivided into primary and secondary collateral systems, illustrated in Figure 1 (24). Primary collaterals refer to arteries of the Circle of Willis that connect the anterior and posterior circulation of the brain (24). Specifically, the Circle of Willis is a ring-like structure that connects the left and right anterior cerebral arteries (ACA) *via* the anterior communicating artery, and the middle cerebral arteries (MCA) and posterior cerebral arteries (PCA) *via* the posterior communicating arteries (Figure 1A) (25). The secondary collaterals refer to the leptomeningeal arteries, which form anastomosis between the distal segments of ACA and MCA, and between the PCA and MCA (Figure 1B) (6).

### Pathophysiology of Cerebral Collateral Recruitment

The pathophysiology of cerebral collateral recruitment in ischemic stroke is not well understood in humans, and has primarily been described in animal models (26). Investigators propose that the extent and timing of cerebral collateral circulation recruitment are crucial factors, and may be broadly dependent on the following factors: the anatomical variations in collateral vessels, the capacity of blood vessels to vasodilate in response to ischemia, and individual risk factors (13, 24).

#### The Anatomical Variations in Collateral Vessels

The recruitment of primary and secondary collaterals depends on natural anatomical variations, size, number, and distribution of vessels. In response to a large vessel occlusion, the primary collateral circulation system provides immediate diversion of blood flow to the ischemic region primarily through the anterior and posterior communicating arteries within the Circle of Willis (24). The involvement of these primary collaterals depends on the natural, anatomical variations in the Circle of Willis. Using magnetic resonance angiography (MRA) in 874 men and 990 women, Hindenes et al. found 47 unique variations in the Circle of Willis, where the absence of the posterior communicating arteries is the most common variation (27). This is clinically relevant, as acute MCA occlusions in patients with a present ipsilateral posterior communicating artery are more likely to have favorable functional outcomes (i.e., modified Rankin score <2) after successful endovascular mechanical thrombectomy, compared to those without an ipsilateral posterior communicating artery (28). The recruitment of leptomeningeal collaterals is hypothesized to occur when the primary collaterals have failed to provide adequate blood flow to the ischemic regions (24), since leptomeningeal arteries are primarily observable in the later phases of ischemia (29). Anatomical variations in the presence of leptomeningeal collaterals have also been investigated by numerous authors using evidence ranging from human cadaver brain dissections to advanced brain imaging, with high inter-individual variability in the number and size of the leptomeningeal arteries (29).

#### The Capacity of Collateral Vessels to Vasodilate

The recruitment of collaterals may also depend on the capacity of the cerebral vasculature to vasodilate in response to ischemic stress through endothelial and metabolic mechanisms (26, 30). Animal studies have demonstrated the importance of endothelial function (i.e., the ability of endothelial cells to produce vasodilatory factors, such as nitric oxide) for collateral recruitment (26). Investigators have inhibited the production of endothelial-derived nitric oxide in rats, which has been shown to reduce cerebral collateral recruitment during ischemia and lead to subsequent larger infarct sizes (31). Additionally, inhalation of nitric oxide during ischemia, a known cerebrovascular vasodilator, can promote cerebral collateral recruitment and reduce infarct size in rats (32). In humans, assessing the association between endothelial function and collateral recruitment in response to an acute ischemic stroke is complex due to the priority for establishing rapid revascularization via administration of hyperacute therapies. Inferences can be made from assessing collateral recruitment of acute stroke patients with chronic small vessel disease, a patient group where poor endothelial function is well-established (33). Lin et al. examined the association between collateral recruitment and small vessel disease in 100 acute ischemic stroke patients who received mechanical thrombectomy (30). Participants who were categorized as having small vessel disease (i.e., presence of severe white matter hyperintensities, lacunar strokes, microbleeds, or enlarged perivascular space) were twice as likely to have poor cerebral collateral circulation (30). The authors postulated that increased stiffness of arterioles, including leptomeningeal arteries, associated with small vessels disease may lead to impaired endothelial function of leptomeningeal collaterals (30). In support of this finding, Giurgiutiu et al. studied 73 ischemic stroke survivors and found that greater white matter hyperintensity volumes were independently associated with poor collateral circulation (34). Mark et al. reported similar findings when examining 178 stroke survivors who received mechanical thrombectomy, where more extensive white matter hyperintensities were independently associated with poor collateral circulation (35).

Given that the vasodilatory responses in cerebral circulation occurs from large arteries to the microcirculation, inferences can also made about collateral vessel recruitment by examining the evidence of the endothelial function of the larger arteries, such as the MCA, or arteries in the Circle of Willis (36). For instance, the recruitment of primary collaterals may depend on their vasodilatory capacity (37). Kim et al. used transcranial Doppler ultrasonography (TCD) to measure flow diversion in the ipsilateral ACA and PCA within 24 h of clinical angiography in 51 patients with acute M1 MCA stenosis or occlusion, and found that 47% of patient had adequate flow diversion (i.e., >30% greater flow velocity compared to contralesionally side) and were associated with recruitment of leptomeningeal collaterals (38). Impaired endothelial function assessed at the MCA or basilar artery is more commonly observed in stroke survivors when compared to healthy age-matched controls, likely due to combined effects of pre-stroke vascular risk (i.e., chronic hypertension) and stroke-related tissue injury (39). Therefore, endothelial function of the MCA post-stroke may be a surrogate measure of collateral circulation recruitment in ischemic stroke. However, the evidence relating endothelial function of the MCA in subacute or chronic phases of stroke and collateral circulation recruitment in response to ischemia are conflicting (40–43). For instance, Hofmeijer et al. investigated the association between collateral circulation and endothelial function using TCD at the MCA in 70 patients with symptomatic carotid artery stenosis (40). The authors found that poor endothelial function at the MCA was associated with the recruitment of collateral circulation via the ophthalmic (TCD) or leptomeningeal arteries (angiography) on the symptomatic hemisphere (40), which may be counter-intuitive. To explain this observation, investigators have postulated that the presence of angiogenic adaptations *via* the development of collateral arteries may be a response to post-stroke ischemic injury and chronic hypoperfusion (24). Therefore, these studies may not sufficiently reflect the association between endothelial function and collateral recruitment in the setting of an acute ischemic stroke, and further investigation is needed.

### Individual Risk Factors for Poor Collateral Recruitment

Older age and modifiable risk factors have been identified as potential individual risk factors for poor collateral circulation (13, 24, 29, 44). In animals, aging is associated with a reduction in the size, number, and diameter of leptomeningeal collateral arteries (45), a process that is also thought to occur in humans (13, 24). Bullitt et al. assessed cerebrovascular structures using MRA in 100 healthy human adults, and found an age-related decline in the number of small vessels (<1 mm diameter) and an abnormal increase in the level of curvature in vessels (46). How these finding may applied to leptomeningeal arteries, however, was not specified. In addition to older age, Menon et al. examined the collateral circulation status of 206 consecutive stroke patients with MCA occlusions, and found that poor collateral circulation status was independently associated with metabolic syndrome (i.e., having three or more of the following: high triglycerides, low high-density lipoprotein cholesterol, high plasma glucose, high blood pressure or medication to control blood pressure, or obesity) (16). Fujita et al. examined the association between chronic hypertension (defined as pre-stroke hypertension diagnosis or use of antihypertensive medications) and collateral circulation in 100 acute ischemic stroke patients, and found that chronic hypertension was associated with poorer collateral recruitment (47). A meta-analysis by Malhotra et al. including nine studies investigated the association between pre-stroke statin treatment, collateral circulation, and infarct size, and found that pre-stroke statin treatment was associated with smaller infarct size, and inconclusive association with collateral circulation status (48). Overall, these studies identified potential individual, modifiable risk factors associated with poor collateral circulation.

## Physical Activity and Its Potential Association With Cerebral Collateral Recruitment

**Figure 2:**
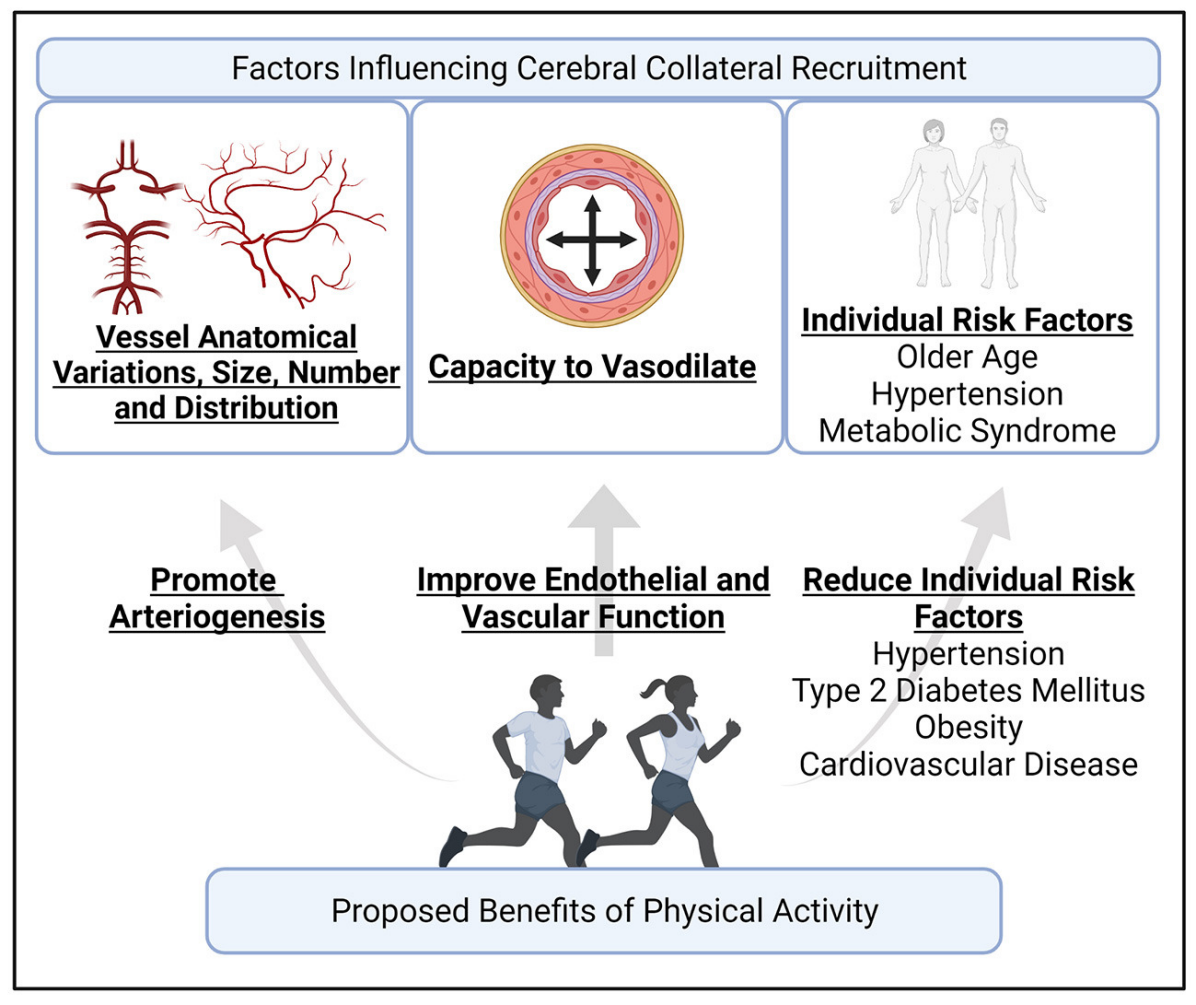
An illustration of the proposed factors influence cerebral collateral recruitment, and how the benefits of physical activity may contribute to favorable collateral circulation recruitment. Cerebral collateral circulation recruitment depends on individual anatomical variations, number, size, and distribution of blood vessel, the capacity of blood vessels to vasodilate, and individual risk factors, such as age (13, 24). Physical activity can potentially contribute to arteriogenesis, including leptomeningeal arteries (52, 58), can improve cerebral endothelial and vascular function (56), and is well-documented in reducing the risk of hypertension, type 2 diabetes mellitus, obesity, and cardiovascular disease (59). Created with BioRender.com.

Physical activity is defined as “any bodily movement produced by skeletal muscle that results in energy expenditure,” and can be sub-typed as leisure-time, household, work, and transportation (49). Exercise is a specific subtype of leisure-time physical activity, which involves planned, structured, and repetitive bodily movements with the purpose of improving or maintaining one of more components of physical fitness, such as cardiorespiratory fitness or muscle strength (49). Exercise can also be further sub-categorized as aerobic or resistance exercise. Aerobic exercise is defined as exercise that involves large muscle groups that can be maintained continuously in a rhythmic nature (50). Resistance exercise is defined as exercise involving periodic bodily movements where external weights provide progressive overload to increase skeletal muscles strength and mass (51). The following section will discuss various cerebrovascular responses to physical activity, primarily aerobic exercise, that may influence collateral circulation. In Table 1 and Figure 2, we summarized the factors influencing cerebral collateral circulation and proposed benefits of physical activity that may improve cerebral collateral circulation.

**Table 1:** Potential association between physical activity and cerebral collateral circulation recruitment.

**Factors influencing cerebral collateral circulation**	**Proposed benefits of physical activity**
Collateral vessel anatomical variations, size, number, and distribution (27, 29)	Promoting growth of new arteriole blood vessels *via* vascular endothelial growth factors (52, 53)
Capacity to vasodilate (30) • Endothelial function and nitric oxide bioavailability (26)	Cerebrovascular adaptations • Increase nitric oxide bioavailability (54, 55) • Increased cerebral blood flow and reactivity (56)
Individual risk factors • Older age (13, 29) • Metabolic syndrome (16) • Hypertension (47) • Pre-stroke statin treatment (48)	Reduce individual risk factors (57) • Hypertension • Type 2 diabetes • Cardiovascular disease • Obesity

### Cardiovascular Response to Physical Activity

The cardiovascular response to physical activity is highly complex and has been extensively described (58, 60, 61). Briefly, the onset of physical activity causes heart rate and stroke volume to increase, resulting in increased cardiac output to meet the increased metabolic demands of contracting skeletal muscles (60). Systolic blood pressure increases with increasing intensity of physical activity, while diastolic blood pressure remains stable or may even decrease (62). Specifically to cerebral circulation, physical activity acutely increases cerebral blood flow with greater increases with higher intensity physical activity (63). This increase in blood pressure and blood flow results in two broad types of interactions at arterial walls to further increase in blood flow through vasodilation: circumference strain and sheer stress (58). Circumference stress occurs when increases in blood pressure stretch and strain the circumference of arterial walls. Increased exposure to circumferential strain on endothelial cells triggers the production and upregulation of endothelial factors, such as nitric oxide. Furthermore, this circumferential strain also stretches the vascular smooth muscles, and subsequently, induces contraction of the smooth muscles to further increase cerebral blood flow. The second type of interaction is called shear stress, where increased blood flow results in parallel forces applied by the blood along the vessel endothelium, which also stimulates the production of endothelial factors, such as nitric oxide. Among numerous factors, this production and upregulation of nitric oxide from these two interactions relaxes vascular smooth muscles, resulting in acute vasodilation and increased blood flow. In addition to nitric oxide, this circumferential strain and shear stress also induces the production of vascular endothelial growth factors, responsible for arteriole and capillary growth (i.e., angiogenesis), with the purpose of providing more blood flow to the required tissues (58, 64).

### Cerebrovascular Adaptations to Physical Activity

Aging is associated with endothelial dysfunction, arterial stiffening, impaired angiogenesis, and risk of cardiovascular disease (65–67). While mechanisms behind these processes are complex and not fully understood, the reduction in bioavailability (i.e., production and release) of nitric oxide with age in humans may be a key link (66, 67). Various measures of cerebral endothelial and vascular function also decline with age, including reduction in global and regional cerebral blood flow and cerebrovascular reactivity (i.e., capacity of blood vessels to response to physiological stress) (68). Wu et al. cross-sectionally assessed global cerebral blood flow using MRI and transcranial 4D flow imaging in 82 people ranging between 7.2 months and 60.7 years old, and found that cerebral blood flow declines with age, starting at 18 years old (69). Similarly, Miller et al. cross-sectionally assessed cerebrovascular reactivity in 39 participants between the age 21-67 years old using MRI and transcranial 4D flow imaging, and found that older participants had lower cerebrovascular reactivity compared with younger participants in global and individual vessel blood flow in the MCA, intracranial artery and basilar artery (70). This age-related decline in blood flow and cerebrovascular reactivity may be associated with poor collateral recruitment observed in older ischemic stroke patients previously discussed.

Life-long physical activity in humans may prevent age-related decreases in nitric oxide bioavailability. Nyberg et al. assessed bioavailability of nitric oxide in 32 people of various age and physical activity levels, and found that older, sedentary participants had reduced nitric oxide bioavailability compared with active, older participants (71). Based on these findings, physical activity is regarded as a potential strategy to increase nitric oxide bioavailability (54, 55). A recent review conducted by Facioli et al. included 16 studies and found that various exercise types (aerobic, resistance, Pilates, Tai Chi), intensities and durations of exercise interventions were associated with increased production of nitric oxide in people with hypertension (72). The effects of physical activity may also translate to improvements in multiple measures of cerebrovascular function. Smith et al. (56) conducted a systematic review and meta-analysis including 34 studies and examined the association between cardiorespiratory fitness (i.e., objectively measured peak oxygen [VO_2peak_] consumption during a graded exercises test) and cerebrovascular function, and the effect of exercise training on cerebrovascular function in healthy and clinical populations. In this review, cerebrovascular function was quantified as cerebral blood flow, reactivity (i.e., change in MCA blood flow given a change in hypercapnic conditions), and resistance (i.e., ratio of mean arterial pressure to cerebral blood flow). The authors found that higher cardiorespiratory fitness was associated with improved cerebrovascular resistance and reactivity compared to lower fitness. However, only participants who were categorized as extreme levels of high cardiorespiratory fitness were associated with higher cerebral blood flow compared with lower fitness levels. In two studies, improvements in cerebrovascular reactivity were associated with corresponding increases in cardiorespiratory fitness after taking part in an aerobic exercise interventions (73, 74). One study reported improvements in cerebrovascular reactivity in healthy older adults using a combined aerobic and resistance training exercise program (75). However, these results were inconsistent in three other studies including stroke, breast cancer, and chronic kidney disease survivors using aerobic exercise training (73, 76, 77). While meta-analysis results suggested that exercise interventions were not associated with changes in global cerebral blood flow, improvements in cerebral blood flow to specific regions (hippocampus and anterior cingulate cortex) were reported by some authors (78–80), but there results were inconsistent (81, 82). The authors highlight the results may have been influenced by heterogenous exercise intervention designs, including intervention lengths (2-12 months), exercise types, and lack of reporting on attendance and adherence to intervention training (56). Taken together, the authors concluded that their findings suggested that higher cardiorespiratory fitness with life-long exercise is associated with improved cerebrovascular function, and enhances our understanding of exercise as a potential approach to decrease age-related changes in cerebrovascular function (56). Higher quality, randomized controlled trials are needed to conclusively determine the benefits of physical activity intervention on cerebrovascular function, as well as determining exact duration, intensity, type and timing of interventions (56). Overall, this evidence highlights the potential effect of physical activity on improved capacity for vasodilation associated with collaterals recruitment.

Physical activity can potentially influence the growth of new arteriole blood vessels (i.e., arteriogenesis) through the production and upregulation of vascular endothelial growth factors (52, 58). Vital et al. conducted a systematic review examining the effect of exercise interventions on peripheral concentrations of vascular endothelial growth factors in humans (83). Of the 10 studies included, four studies (two randomized controlled and two non-randomized controlled trials) reported an increase in vascular endothelial growth factors in various elderly populations after the interventions, primarily involving aerobic exercise (83). While the six remaining studies (four randomized controlled, one non-randomized controlled, and one randomized uncontrolled trial) found no change in vascular endothelial growth factor concentrations, the heterogeneity in type of physical activity, duration and length included across interventions made it difficult to effectively determine optimal dose of exercise (83). Boyne et al. examined 16 chronic stroke survivors and found an the acute increase in circulating vascular endothelial growth factors after a single bout of high intensity interval treadmill training (84). However, visible changes in vasculature in cerebral collateral circulation as a result of physical activity has primarily been demonstrated in animal models (52). One known human, cross-sectional study conducted by Bullitt et al. examined the association between self-reported physical activity and cerebral vascular structure (i.e., vessel number, average vessel radius, and vessel shape) of the MCA, ACA, and PCA assessed using MRA in 14 healthy older adults (53). The authors found that participants categorized as “high activity” (i.e., engaged in regular aerobic activities for at least 180 min per week for the past 10 years) trended toward having greater number of small vessels (<1 mm diameter), but not larger diameter vessels, and smoother vessel curvature compared to inactive participants (53). While not mentioned by the authors, these greater number of small vessels associated with higher physical activity levels may also include leptomeningeal arteries. The authors were unable to detect arteries smaller than 0.5 diameter due to limitations in voxel size in MRA techniques, potentially excluding the leptomeningeal arteries (53). Overall, this provides promising evidence that physical activity may help prevent age-related attrition in collateral arteries in humans.

### Physical Activity Guidelines and Individual Risk Factors

The wide range of physical and mental health benefits of physical activity are well-documented and evidenced by numerous systematic reviews and meta-analyses (59, 85–89). In particular, Warburton et al. conducted a comprehensive systematic review that included 254 articles, primarily observational studies, highlighting consistent evidence for the independent association between physical activity and reduced risk of all-cause mortality and prevalence for a variety of chronic diseases, such as cardiovascular disease, hypertension, stroke, obesity, and type II diabetes mellitus (57). Numerous additional meta-analyses of randomized controlled trials have also reported that physical activity interventions are effective in the prevention and treatment of hypertension (54 trials, 2,419 participants) (90), type 2 diabetes mellitus (11 trials, 846 participants) (91), and favorable modification of numerous cardiometabolic biomarkers (160 trials, 7,487 participants), including lipid profiles, fasting insulin, and glycosylated hemoglobin A1c (92). As such, the American Heart Foundation and American College of Sports Medicine recommend older adults to engage in at least 30-min of moderate-to-vigorous physical activity per day to prevent and manage chronic diseases (93). The American Heart Association Stroke Council have similar recommendations for stroke survivors to improve physical function and reduce risk factors for stroke for secondary prevention (94). Given these benefits, physical activity may help reduce individual risk factors associated with poor collateral circulation.

## Studies Directly Assessing the Association Between Physical Activity and Cerebral Collateral Circulation

The effect of physical activity on cerebral collateral circulation has been studied primarily in animals. Rzechorzek et al. showed that voluntary wheel-running in mice (i.e., equivalent to incidental physical activity in humans) reduced age-related decrease cerebral collateral artery diameter, length, and number, and infarct size after middle cerebral artery occlusion, compared to sedentary mice (95). Furthermore, these adaptions were associated with upregulation of endothelial nitric oxide (95). No identified studies have evaluated the association between pre-stroke physical activity and cerebral collateral circulation in human acute ischemic stroke. Although the association between nitric oxide and cerebral collateral circulation in human ischemic stroke remains unclear, one of the mechanisms underlying the potential effects of pre-stroke statin treatment on collateral circulation in humans includes the upregulation of endothelial nitric oxide (48). However, the effect of physical activity interventions on coronary collateral circulation in people with coronary artery disease has been examined (96). Nickolay et al. summarized the evidence from seven studies that examined the effect of aerobic exercise interventions to increase coronary collateral flow in humans, and revealed consistent evidence that aerobic exercise is beneficial in promoting coronary collateral circulation, using angiography or the Collateral Flow Index (i.e., considered the “gold standard” for assessing coronary collateral flow) (96). In particular, Möbius-Winkler et al. randomized 60 patients with severe coronary artery disease to high intensity or moderate intensity aerobic exercise training, and showed increased coronary collateral circulation compared with usual care patients using Collateral Flow Index and angiographic evidence (97). Stoller et al. examined muscle tissue in 110 patients undergoing diagnostic coronary angiography, and found that patients with greater self-reported leisure-time physical activity levels had greater femoral artery collateral circulation (98). While no studies have been conducted in humans, these studies provide evidence in mammalian models to motivate further investigation in the associations between cerebral collateral circulation and physical activity in human acute ischemic stroke.

## Measuring Cerebral Collateral Circulation

Measurement considerations for cerebral collateral circulation are summarized in Table 2. The conventional method of evaluating the collateral circulation status in acute ischemic stroke involves using visual grading systems, often assessing the presence, extent and/or timing of collateral vessels (namely leptomeningeal arteries) relative to the ischemic region (99). Different collateral recruitment grading systems have been developed using brain imaging to visual collateral vessels (99). The most commonly used grading systems (106) are those developed by the American Society of Interventional and therapeutic Neuroradiology and Society of Interventional Radiology (ASITN/SIR) (107), Alberta Stroke Program Early CT (ASPECT) Score for collaterals (108), Christoforidis et al. (109, 110), and Miteff et al. (111). Seker et al. compared these four grading systems using dynamic CTA, and found that ASITN/SIR and the ASPECT score to be superior to Christoforidis and Miteff systems in predicting infarct volumes in 30 acute stroke patients with M1 or terminal carotid artery occlusions (106). Furthermore, ASITN/SIR grading system has been used by multiple investigators to predict stroke outcomes in acute ischemic stroke patients, where favorable collateral grading has been associated with smaller infarct core volume and favorable functional outcomes (8, 112, 113).

**Table 2:** Measurement considerations for cerebral collateral circulation and pre-stroke physical activity.

**Outcome**	**Methodology**	**Strengths**	**Limitations**
Cerebral collateral circulation	Visual grading scales using angiography (DSA, MRI, CTA, CTP) (99)	“Gold Standard” (namely DSA) (13)	Requires expert input (100)
	Hypoperfusion intensity ratio (101)	Automated; requires minimal expert input (100)	Indirect measure (101)
	Transcranial Doppler (99)	Can be completed post-stroke (40–43)	Unclear clinical relevance to acute ischemic stroke (13)
Pre-stroke physical activity	Self-report questionnaires	• Suitable of retrospective and case-control studies (17) • Inexpensive and easy to administer (102)	Risk of recall bias (102, 103)
	Objective measurements (accelerometers and pedometers)	• Suitable for prospective cohort studies • Reduced risk of bias and improve accuracy (104)	Expensive and time consuming (13, 105)

*CTA, computed tomography angiography; CTP, computed tomography perfusion; DSA, digital subtraction angiography; MRI, magnetic resonance imaging*.

The “gold standard” imaging modality to visualize cerebral collateral circulation in ischemic stroke is digital subtraction angiography (13, 114). However, digital subtraction angiography is invasive and costly, so other methods may be preferred (13, 114, 115). Furthermore, in current clinical practice, the imperative for rapid revascularisation means that a full set of angiographic images, with injection of multiple arteries, is not obtained prior to treatment. This limits the assessment of collateral flow largely to MCA occlusion. The anterior cerebral, but not posterior cerebral, collateral pathways can be assessed based on an internal carotid artery injection. However, non-invasive multi-modal brain imaging techniques performed as part of standard clinical care for acute stroke provide numerous opportunities for cerebral collateral circulation assessment, including MRI, MRA, computed tomography angiography (CTA) and perfusion (CTP) (13, 99, 100, 116). For MRI, fluid-attenuated inversion-recovery sequences can be used to visualize “vascular hyperintensities” as an indirect method of assessing collateral circulation in acute ischemic stroke (117). Single- and multi-phase CTA and/or MRA are often used to identify the presence of large vessel occlusion, and can also be used to assess collateral vessels (13, 100). In addition to providing invaluable information for the selection of patients for thrombolytic and endovascular therapy (118–122), CTP offers multiple opportunities for collateral circulation assessment (100). With post-processing software, source images from CTP (maximum intensity projections) can be used to reconstruct four dimensional CTA to assess collateral circulation (123). Time-to-maximum (Tmax) is a perfusion parameter derived from deconvolution with an arterial input function, indicates the severity of blood flow delay which, in the context of arterial occlusion, reflects collateral blood flow quality (124). Regions of brain with perfusion delay defined as Tmax > 6 s provides an estimate of the tissue at risk (ischemic core and penumbra) (101, 125). The hypoperfusion intensity ratio (HIR), defined as the volumetric ratio of tissue with Tmax > 10 s to Tmax >6 s, can serve as an indirect estimate of collateral circulation status (101, 125). The HIR has been well correlated with collateral grading in acute ischemic stroke patients as assessed by digital subtraction angiography by multiple investigators (8, 112, 126). Other CTP parameters have been correlated with collateral circulation, including cerebral blood volume (CBV) and cerebral blood flow (CBF) (105, 127). Specifically, higher relative CBV (rCBV), defined as the ratio of mean CBV values within the Tmax > 6 s regions to mean CBV values within the Tmax ≤4 s regions (normal brain tissue), has been correlated with favorable collateral grading in acute ischemic stroke patients (105). Using CBF <30% to estimate the ischemic core volume, slower baseline ischemic core-growth rate, defined as the ischemic core volume divided by the time between stroke onset and CTP imaging, has been correlated with favorable collateral grading in acute ischemic stroke patients (127). Finally, while not routinely assessed, TCD can be used to measure flow velocities and collateral circulation (41, 128). Specifically, TCD has been used to measure flow diversion in the ipsilateral anterior and posterior communicating arteries (i.e., >30% higher than contralateral flow velocity) within 24 h of clinical angiography in acute M1 MCA stenosis or occlusion, and has been associated with recruitment of leptomeningeal collaterals as seen on digital subtraction angiography (38). TCD can also be used to measure collateral flow at the ophthalmic arteries (40, 41).

## Measuring Pre-Stroke Physical Activity Levels

Measurement considerations for pre-stroke physical activity are summarized in Table 2. Measuring pre-stroke physical activity is challenging with inherent limitations based on study design (103, 129). Based on a recent systematic review we conducted, along with other studies measuring pre-stroke physical activity (23, 130–132), most studies were retrospective and case-control designs and relied on self-reported physical activity questionnaires to recall pre-stroke activities (17, 18). Questionnaires are relatively inexpensive, low resource burden, convenient to administer, and ideal for clinical settings (102). However, they are prone to risk of recall and social desirability bias, leading to questionable accuracy, especially for incidental, unstructured physical activity (102). Assessing pre-stroke physical activity soon after stroke incidence (i.e., during their acute hospital stay) may reduce potential recall bias associated with memory. However, additional challenges may present in acute stroke patients including impaired cognition and memory, aphasia, and additional social desirability factors associated with self-image after stroke (103). Prospective cohort studies have the capacity to include objective measurements of physical activity, including accelerometers and pedometers, to reduce risk of bias and improve accuracy of physical activity measurement (104). However, prospective cohort designs are often expensive and time-consuming due to the long follow-up and large samples required for disease incidence (103, 133). As such, few prospective cohort studies have been conducted, including the Physician's Health Study (132) and Women's Health Initiative cohorts (23), but neither studies included objective measures of physical activity. Nevertheless, self-reported questionnaires remains a feasible, cost-effective and pragmatic method to measure pre-stroke physical activity in acute or subacute settings, as demonstrated by many investigators (17, 18).

Specific to acute clinical settings, assessing pre-stroke physical activity can be challenging. The selection of measurement tools need to consider the balance between burden of administering the questionnaire (i.e., time and attentional resources of patients and healthcare providers) and the level of detail on type and dose (i.e., intensity, frequency, and time) of pre-stroke physical activity (102). A variety of pre-stroke physical activity questionnaires have been used in the literature (17, 18). Authors have used measures that are relatively less time consuming, such as single-questionnaire measures to assess if they meet minimum duration and frequency of leisure-time physical activity (134–137) or standardized questionnaires [i.e., Saltin-Grimby Physical Activity Level Scales (138)]. However, the brief nature of these questionnaires may provide limited detail on physical activity type and dose and may preclude further discussion on how to modify physical activity after stroke. Authors have also used relatively longer and more detailed physical activity quesitonnaires (130, 139, 140), such as the International Physical Activity Questionnaire (141) and the Physical Activity Scale for the Elderly (142). These standardized questionnaires provide more information of physical activity type and dose, and may also be useful in continued assessment and physical activity counseling post-stroke. Furthermore, the selection of questionnaires needs to be age-appropriate, where the types of physical activities assessed should be those commonly engaged by older adults (17).

## Discussion

### Potential Barriers and Solutions to Measuring Collateral Circulation

Given the well-documented benefits of physical activity and potential positive influence on cerebral vascular function discussed above, we hypothesize that greater pre-stroke physical activity is associated with better collateral circulation in ischemic stroke. Assessing pre-stroke, and post-stroke, physical activity in acute and subacute clinical settings is feasible, and the advent of advanced brain imaging integrated into routine clinical care offers a cost-effective opportunity to assess of collateral circulation in acute ischemic stroke. However, collateral circulation grading using advanced imaging modalities often requires expert input from experienced neurologists and radiologists, and potential post-processing techniques by imaging scientists (100). This may be a barrier for collateral circulation grading where such expertise is limited within clinical and research settings. The HIR and rCBV may be solutions to this barrier, as perfusion maps that include Tmax and CBV parameters and associated HIR and rCBV values can be generated by multiple automated software, such as the RApid Processing of Perfusion and Diffusion (RAPID) software, without extensive and ongoing expert input (100, 101, 105). Clinically relevant HIR threshold values have been established to reflect favorable or unfavorable collateral status. Olivot et al. examined 99 acute ischemic stroke patients, and reported that an HIR ≥ 0.4 was associated with greater infarct growth and final infarct volume, greater admission stroke severity, and unfavorable functional outcomes after stroke (8). Similar thresholds have been reported by other authors, where favorable HIR has been associated with less infarct growth, favorable functional outcomes, and even potentially eligibility for thrombectomy (112, 143–145). Arenillas et al. examined 158 acute ischemic stroke patients, and reported that higher rCBV was associated with favorable collateral circulation and slower infarct growth at 27-h follow-up (105), and favorable functional outcome in patients after acute ischemic stroke (105). Furthermore, Cortijo et al. invested 100 acute ischemic stroke patietns, and reported that lower rCBV was associated with poor colleateral circulation, and poor functional outcome in the absence of recanalisation (146). Overall, both HIR and rCBV serve as accessible opportunities for a wider group of health professions (i.e., physiotherapists and exercise physiologists) and research communities to indirectly assess collateral circulation status with relatively minimal on-going expert input. However, in the absence of acute ischemic stroke and clinical imaging, in the case of potential clinical trials, follow-up assessments to evaluate the change in collateral circulation will require expert input, as HIR and rCBV has not been assessed outside of the hyperacute phases of stroke. Advanced MRA may potentially be used to visualize collateral vessels (13), and TCD measurements at the MCA, ACA, or PCA in response to forced-dilatory responses (i.e., hypercapnic breathing maneuvers) may be used as surrogate measure of collateral recruitment (40–43). However, further investigation is required regarding the clinical relevance of cerebrovascular function assessments conducted post-hyperacute stroke phase (13).

### Physical Activity, Collateral Circulation, and Post-stroke Recovery

Physical activity is a widely accepted and important strategy for the prevention and management of stroke. Notably, physical activity is currently recommended to improve physical function and manage vascular risk factors for secondary prevention in people who have had a stroke (94). However, to improve our understanding on the role of physical activity on key stroke recovery outcomes, generating new information on the possible link between physical activity and collateral circulation is a worthwhile endeavor. The Stroke Recovery and Rehabilitation Roundtable regard clinically acquired CT imaging, including perfusion, in the hyperacute phase of stroke as a potential biomarker for stroke recovery and a developmental priority for future research (147). Exercise and physical activity interventions are effective and recommended for reducing disability, and improving mobility and physical function in stroke survivors (148, 149). If physical activity and collateral circulation are associated, collateral circulation status may emerge as a biomarker to stratify or identify patients for clinical trials, such that we can target patients with poor collateral status and suspected poor functional outcomes who would most benefit from enrolling in physical activity interventions. These physical activity interventions can potentially reduce the risk of poor clinical outcomes in the event of first-ever strokes for high-risk populations or recurrent strokes for stroke survivors through improvements in cerebral collateral circulation. Furthermore, physical activity interventions have the potential to improve functional recovery after stroke. Limaye et al. conducted a systematic review examining the effect of aerobic exercise interventions on serum biomarkers of neuroplasticity in human stroke survivors (150). The authors included nine studies, and found that aerobic interventions can increase brain-derived neurotrophic factors, insulin-like growth factor 1, and VEGF. However, further investigation is needed to discern how these serum biomarkers of neuroplasticity are associated with collateral circulation, the optimal exercise type and dose, most appropriate timing to commence physical activity interventions after stroke (i.e., how early), and the effect on functional recovery across different phases (i.e., acute, subacute and chronic) post-stroke in humans (148, 150).

The association between cerebral collateral circulation and cardiorespiratory fitness has also not been examined. While physical activity is closely associated with cardiorespiratory fitness (151), cardiorespiratory fitness is a direct physiological measurement associated with endothelial function, and potentially provides more accurate prediction cardiovascular disease risk compared with physical activity alone (152). However, assessment of cardiorespiratory fitness is relatively more burdensome for patients and requires specialized equipment and trained personal to conduct (153). While cardiorespiratory fitness is related to cerebrovascular function (56), further investigation is needed to explore the association between cardiorespiratory fitness and cerebral collateral circulation.

## Conclusion

Cerebral collateral circulation has physiological and clinical prognostic value and may be an important therapeutic target for acute ischemic stroke survivors. While the effect of physical activity on cerebrovascular function has been investigated, the association between physical activity and cerebral collateral circulation is unknown. Future studies can take advantage of routinely acquired medical imaging in primary stroke centers to assess collateral circulation status, its association with physical activity, and potential utility as a biomarker to identify patients who would benefit from physical activity and stroke recovery interventions.

## Data Availability Statement

The original contributions presented in the study are included in the article/supplementary material, further inquiries can be directed to the corresponding author/s.

## Author Contributions

SH led the manuscript and designed, conceptualized, and drafted the manuscript. SK, EW, BC, and AB played major roles in designing, conceptualizing, and revising the manuscript for intellectual content. All authors contributed to the article and approved the submitted version.

## Conflict of Interest

The authors declare that the manuscript and research was conducted in the absence of any commercial or financial relationships that could be construed as a potential conflict of interest.

## Publisher's Note

All claims expressed in this article are solely those of the authors and do not necessarily represent those of their affiliated organizations, or those of the publisher, the editors and the reviewers. Any product that may be evaluated in this article, or claim that may be made by its manufacturer, is not guaranteed or endorsed by the publisher.
